# Antimicrobial and lipase inhibition of essential oil and solvent extracts of *Cota tinctoria* var. *tinctoria* and characterization of the essential oil

**DOI:** 10.55730/1300-0527.3430

**Published:** 2022-04-14

**Authors:** İshak ERİK, Gözde BOZDAL, Sıla Özlem SENER, Büşra KORKMAZ, Şengül Alpay KARAOĞLU, Salih TERZIOĞLU, Nurettin YAYLI

**Affiliations:** 1 Department of Pharmacognosy, Faculty of Pharmacy, Karadeniz Technical University, Trabzon, Turkey; 2 Department of Botany, Faculty of Pharmacy, Karadeniz Technical University, Trabzon, Turkey; 3 Department of Biology, Faculty of Science, Recep Tayyip Erdoğan University, Rize, Turkey; 4 Department of Forest Botany, Faculty of Forestry, Karadeniz Technical University, Trabzon, Turkey

**Keywords:** Antimicrobial, *Cota tinctoria* var*. tinctoria*, GC-FID/MS, lipase inhibition, volatile compounds

## Abstract

The essential oil (EO) of *Cota tinctoria* var. *tinctoria* was analyzed using GC-FID / MS. A total of 51 compounds were determined from this taxon, accounting for 99.79% in hydrodistillation. Monoterpenes were the primary chemical class for the volatile organic compounds in the EO (36.1%, 13 compounds). Borneol (18.1%), camphor (14.9%), and ***β***-pinene (11.3%) were the major components in the EO of *C. tinctoria* var. *tinctoria*. The antimicrobial activities of EO and *n-*hexane, acetonitrile, methanol, and water solvent extracts of the taxon were screened in vitro against ten microorganisms. The EO yielded the best activity (15 mm, 372.5 MIC, 59600 μg/μL) against *Mycobacterium smegmatis*. The acetonitrile extract was the most active against the *Staphylococcus aureus* and *Bacillus cereus* with 274 μg/mL MIC value. IC_50_ values for the lipase enzyme inhibitory activity of EO and solvent extracts (*n-*hexane, acetonitrile, methanol, and water) were found to be 59.80 ± 4.3285 μg/mL 68.28 ± 3.1215 μg/mL, 52.60 ± 3.7526 μg/mL, 48.73 ± 2.8265 μg/mL, and 99.50 ± 5.5678 μg/mL, respectively.

## 1. Introduction

In the Flora of Turkey, *Cota* J. Gay was classified as a section in the genus *Anthemis* L. together with the sect. *Anthemis* and sect. *Maruta* (Cass.) Griseb. [1]. The sect. *Cota* was accepted as a genus after changing generic and infrageneric concepts [2–4]. Thus, the studied taxon is formerly known as *Anthemis tinctoria* var. *tinctoria* which has already been reported as synonym of *Cota tinctoria* var. *tinctoria* [5]. All *Cota* taxa are mainly distributed in North Africa, Caucasia, Europe (except north of Europe), and Central Asia [6,7]. The genus *Cota* includes up to 17 species and 20 taxa, and 9 of them are endemics [8].

Several *Anthemis* species have been used as a traditional medicine to treat psoriasis (*A. cotula*) [9], diaphoretic, carminative (*A. nobilis*) [10], abdominal pain, kidney disease (*A. cretica*) [11]. Infusions and decoctions of *A. creatica* ssp. *tenuiloba* and *A. austriaca* have been reported to be used against abdominal pain, hemorrhoids, and colds [12]. It has been reported that *A. wiedemanniana* plant has sedative, antispasmodic effects and is used in urinary problems [13]. It is known that the decoction prepared from the flowers of *C. tinctoria* var. *tinctoria* is used for antidiabetic and antispasmodic purposes [13]. Some *Anthemis* species showed various biological activities such as antioxidant [14], antiproliferative [15], antidiabetic [16], antiprotozoal [17], antispasmodic [18].

According to the literature, even though Anthemideae is one of the most phytochemically investigated tribes of the Compositae, only essential oils of *Anthemis nobilis* L. [19], *A. aciphylla* Boiss. var. *discoidea* Boiss. [20], *A. altissima* L., *A. chia* L., *A. tomentosa* L., *A. weneri* L. ssp. *weneri* Stoj. & Acht., *A. auriculata* Boiss., *A. melanolepis* Boiss., *A. cotula* L. and *A. tinctoria* L. var. *parnassica* [21], *A. hyalina* DC. [22], *A. tinctoria* L. [23], *A. cretica* L. ssp. *leucanthemoides* (Boiss.) Grierson [24], *A. ruthenica* M.B. and *A. arvensis* L. [25], *A. wiedemaniana* Fish. Et. Mey. [26], *A. melampodina* auct. Non Delili [27], *A. carpatica* Willd. [28], *A. xylopoda* O. Schwarz [29], *A. montana* L. ssp. *carpatica* [30], *A. altissima* L. [31], *A. triumfetti* (L.) DC. [32], *A. altissima* L. var. *altissima* [33], *A. cretica* subsp. *messanensis* (Brullo) Giardina & Raimondo, *Anthemis arvensis* L. subsp. *arvensis*, and *A. cretica* subsp. *columnae* (Ten.) Frezén [34], *A. dipsacea*, *A. pectinata* var. *pectinata* and *A. pseudocotula* [19] have been investigated so far.

Obesity is a long-term problem which causes a series of psychological and physiological problems. Many kinds of chronic metabolic diseases are caused by obesity. Porcine pancreatic lipase (PPL) inhibitors have been of interest in obesity treatment research in recent years. PPL inhibitors from natural products have a wide range of sources and low toxicity and have therefore attracted interest. Because of these advantages, they could lead to new health products in the pharmaceutical industry [35].

To the best of our knowledge, total phenolic and flavonoid contents, antioxidant activity, butyrylcholinesterase, acetylcholinesterase, and tyrosinase enzyme inhibition of methanol extract of *C. tinctoria* var. *tinctoria* were mentioned [36]. Cytotoxic activity of ethanol extract of *C. tinctoria* flowers was investigated [37]. In another study, phenolic constituents, cytotoxic activity, and dyeing properties for the ethanol and aqueous extracts of stem, flower, and root of the plant were studied [38]. However, antimicrobial activity and lipase enzyme inhibition for the essential oil and solvent extracts (*n-*hexane, acetonitrile, methanol, and water) of *C. tinctoria* var. *tinctoria* were not studied. Hence, we focused our study on the essential oil composition with GC and GC-MS analysis, antimicrobial and lipase enzyme inhibition activities of the EO and the solvent extracts of *C. tinctoria* var. *tinctoria* were screened in vitro against ten microorganisms and porcine pancreatic lipase, respectively.

## 2. Materials and methods

### 2.1. Plant materials

Aerial part of *C. tinctoria* var*. tinctoria* (160 g, wet) was harvested from Şiran, Gümüşhane at a height of 1650 m in May 2018. The plant was collected by Prof. Nurettin Yaylı and authenticated by Prof. Salih Terzioğlu. Voucher specimen (KATO: 19258) has been deposited in the Herbarium of the Faculty of Forestry, Karadeniz Technical University, Turkey.

### 2.2. Chemicals and reagents

All solvents (methanol, acetonitrile, and *n-*hexane) and chemicals (Tris-HCl and *p*-nitrophenyl butyrate) used were purchased from Sigma-Aldrich in analytical grade.

### 2.3. Hydrodistillation procedure for the obtaining of EO

The aerial part of *C. tinctoria* var*. tinctoria* (106 g, dry) was grounded with plant mill into small pieces and then hydrodistillated (HD) with a modified Clevenger-type apparatus with cooling bath (−15 °C, 3 h), yield (*v/w*): 0.079%. After the HD, EO was extracted with *n-*hexane (0.5 mL) and dried over anhydrous Na_2_SO_4_ and stored in a dark glass bottle in the refrigerator at 4 °C prior to the GC-MS analysis.

### 2.4. Solvent extractions (*n*-hexane, acetonitrile, methanol, and water solvents) of *C. tinctoria* var. *tinctoria*

The aerial parts of the plant (40 g, dry) were blended into small pieces. Blended material (5 g, each) was extracted (25 mL × 3; 12 h each) using maceration method at room temperature with analytical grade *n-*hexane, acetonitrile, methanol, and water solvents in flasks (50 mL) separately. After the suction filtration, the same extracts were combined and solvents were evaporated or lyophilized to yield crude *n-*hexane (0.0722 g), acetonitrile (0.0439), methanol (0.5234 g), and water extract (0.0435 g) [39, 40].

### 2.5. Gas chromatography-mass spectrometry (GC-FID/MS)

GC-MS analysis of the EO was carried out by a Shimadzu QP2010 ultra, having Shimadzu 2010 plus FID, PAL AOC-5000 plus auto sampler and Shimadzu Class-5000 Chromatography Workstation software. Restek Rxi-5MS capillary column (30 mm × 0.25 mm × 0.25 μm) (USA) was used for the analysis. Sample (1 μL, in HPLC grade *n-*hexane) injection was performed in split mode (1:30) at 230 °C. Initial column temperature was 60 °C for 2 min, then increased to 240 °C with a 3 °C/min heating ramp. The final temperature for the oven was held at 250 °C for 4 min. Helium (99.999%) was the carrier gas with 1 mL/min flow rate. MS detection was implemented in electronic impact mode (EI, 70 eV, and scan mode 40–450 *m/z*). Sample was analyzed and mean was reported.

### 2.6. Identification of volatile constituents

RI values of the volatile components in EO of *C. tinctoria* var. *tinctoria* were determined by Kovats method (Table 1) [39–44]. Volatile compounds of the EO were identified by comparisons of RI values with those reported in the literature RI [45–75] and MS data matching mass spectral libraries (NIST, Wiley7NL, FFNSC1.2, and W9N11).

### 2.7. Antimicrobial activity assessment (agar-well diffusion method)

All test microorganisms: *Escherichia coli* ATCC35218, *Yersinia pseudotuberculosis* ATCC911, *Pseudomonas aeruginosa* ATCC43288, *Enterococcus faecalis* ATCC29212, *Staphylococcus aureus* ATCC25923, *Bacillus cereus* 709 Roma, *Mycobacterium smegmatis* ATCC607, *Candida albicans* ATCC60193, *Candida tropicalis* ATCC 13803; and *Saccharomyces cerevisiae* RSKK 251 were obtained from the Refik Saydam Institute of Hygiene and Public Health (Ankara, Turkey). The adapted antimicrobial screening test (agar-well diffusion method) was used earlier [76–77]. Each tested microorganism was suspended in Brain Heart Infusion (BHI) and diluted approximately 10^6^ colony-forming units (per mL), which were “flood-inoculated” onto the surface of BHI agar and Sabouraud Dextrose Agar (SDA), then dried. SDA was used for *C. albicans*. Wells (5 mm diameter) were cut from the agar, and the extracts (100 μL, each) were delivered into the wells. The plates were incubated (35 °C, 18 h) and antimicrobial activity was evaluated by measuring the inhibition zone against the test organism. The EO dissolved in *n-*hexane, and other solvent extracts (acetonitrile, methanol, and water solvents) were dissolved in dimethyl sulphoxide to prepare stock solutions (43.500–523.400 μg/mL). *n-*Hexane and dimethyl sulphoxide were used as solvent control with dilution of 1:2. Ampicillin, streptomycin, and fluconazole were used as positive controls at 10 μg/mL, 10 μg/mL, and 5 μg/mL concentrations, respectively (Table 2).

### 2.8. Agar dilution minimum inhibitory concentration (MIC) assay

After the antimicrobial properties of the EO, *n-*hexane, acetonitrile, methanol, and water extracts of *C. tinctoria* var. *tinctoria* were investigated quantitatively (Table 2), MIC values (μg/mL) were determined [76–77]. The antibacterial and antifungal assays were carried out in Mueller–Hinton broth (MH) (pH 7.3) and buffered yeast nitrogen base (Difco, Detroit, MI) (pH 7.0), respectively. The microdilution test plates were incubated (35 °C, 18 h). Brain heart infusion broth (BHI) (Difco, Detriot, MI) was used for *M. smegmatis*, and incubated at 35 °C (48–72 h). The MIC was the lowest concentration that showed no growth. Ampicillin (10 mg/mL), streptomycin (10 mg/mL), and fluconazole (5 mg/mL) were used as standard, respectively. *n-*Hexane and dimethyl sulphoxide were used as solvent control with dilution of 1:10 [39, 76–77].

### 2.9. Lipase inhibitory effect assay (PPL)

Lipase inhibitory assay for the EO and solvent extracts (*n-*hexane, acetonitrile, methanol, and water) of *C. tinctoria* var*. tinctoria* were studied with the modified method using *p-*nitrophenyl butyrate (*p-*NPB) as substrate [39]. All extracts (25, 100, 200, and 400 μg/mL concentrations) were dissolved with buffer solution (0.1 M Tris-HCl, pH = 8.0) and 0.1% DMSO. Orlistat was used as positive control and prepared as 6.25, 12.5, 25, 50, and 100 μg/mL concentration solutions. The experimental method was designed with A, B, C, and D wells; A: 90 enzyme solution [(Crude porcine PL type II) - (200 units/mL)], 5 μL substrate solution (10 mM p-NPB in acetonitrile); 5 μL buffer solution (0.1 M Tris-HCl buffer, pH = 8.0); B: 90 μL enzyme solution, 10 μL buffer solution; C: 90 μL enzyme, 5 μL sample solution, 5 μL substrate solution; and D: 90 μL enzyme solution], 5 μL sample solution, 5 μL buffer solution. The plates were incubated at 37 °C (15 min) then substrate solution (10 mM *p-*NPB in acetonitrile) was added to each related well which were incubated again at 37 °C (15 min). The absorbance of the solutions was observed at 405 nm in a 96-well microplate using a SpetrostarNano-BMG LABTECH spectrophotometer. Experiments were carried out in triplicate. Results were stated as mean ± standard deviation (SD). Statistical significance level was considered p < 0.05 [78]. The percentage of PPL inhibition was calculated by the following equation: PPL inhibition (%) = [[(A – B) – (C – D)] / (A–B)] × 100. Finally, IC_50_ values for the PPL were calculated graphically [39].

## 3. Results and discussion

### 3.1. EO composition of *C. tinctoria* var. *tinctoria*

Volatile components in the EO of the *C. tinctoria* var. *tinctoria* were analyzed by GC-FID/MS using Rxi-5MS capillary column. Identification of the volatile constituents in EO was made by comparison of RI and MS data with literature [39–75]. The chemical profile of volatiles, the percentage content, and calculated retention indices of the constituents of the taxon are presented in Table 1. Borneol (18.11%), camphor (14.90%), ***β***-pinene (11.26%), camphene (10.69%), eucalyptol (6.67%), valencene (6.37%), and ***α****-*pinene (6.35%) were the major compounds in the EO of *C. tinctoria* var. *tinctoria* (Table 1). Monoterpenes (36.11%) and oxygenated monoterpenes (23.22%) were the main constituents of the EO obtained from aerial parts of *C. tinctoria* var. *tinctoria*., and sesquiterpenes (10.75%) and oxygenated sesquiterpenes (4.62 %) were the second major components in the EO of *C. tinctoria* var. *tinctoria*.

In the literature, the essential oil analyses of *C. tinctoria* plant at different times and regions showed differences in their main components and ratios like borneol (16.0%) and spatulenol (16.0​%) [79]; 1,​8-​cineole (7.9​%)​ and *β-*​pinene (7.3 ​%)​ [80]; *α-*​eudesmol (10.2 ​%)​, and *γ*-​cadinol (8.7​%)​ [81]​; and 1,​8-​cincole (7.9​%)​, and *β* -​ pinene (7.​3 ​%)​ [82]. These results showed the variation for the EO of *C. tinctoria* species in the literature.

A literature review has revealed that phytochemical analysis of *C. tinctoria* var. *tinctoria* had shown lipophilic extract, and GC-MS analysis was mentioned that content was rich in saturated fatty acids [83]. Isolation of 3-glucoside, and 3-rutinoside of patuletin from acetone extract of the leaves of *A. tinctoria* var. *subtinctoria* was reported [84].

In a study, the total flavonoid and phenolic contents, antimicrobial, the antioxidant, antibiofilm activities, and anticholinesterase of *A. stiparum* subsp. *sabulicola* aerial parts methanolic extract and essential oil were reported and 72 constituents (99.02%) were exhibited, and major compounds were determined as germacrene D, *t-*cadinol, camphor, spathulenol, and isoamyl salicylate with percentage of 11.13%, 11.01%, 6.73%, 6.50%, 6.45%, respectively [82]. The volatile constituents of the EOs obtained from the aerial parts of *A. cretica* subsp. *messanensis* (Brullo) Giardina & Raimondo, from the aerial parts of the rock-grown form and the cultivated of *A. arvensis* L. subsp. *arvensis*, and from flowers and leaves of *A. cretica* subsp. *columnae* (Ten.) Frezén were reported.

Torreyol (85.4 %) from *A. arvensis* subsp. *arvensis*, (*E*)-chrysanthenyl acetate (28.8%) from *A. cretica* subsp. *messanensis*, and 18-cineole (13.3% and 12.2%) from both flower and leaf oils of *A. cretica* subsp. *columnae* were reported as main constituents [34]. The essential oil analysis of *A. fungosa* and its antioxidant (IC_50_ = 3000 ± 8.3 μg/mL, compared with the standard, quercetin, with an IC_50_ of 33.3 ± 1.3 μg/mL) and antimicrobial activities against nine microorganisms (six bacteria and three fungi) were found to have strong antimicrobial activity (MIC = 32 μg/mL) against *K. bacteria*, *S. epide*rmidis, and all of the tested fungi [85]. The hepatoprotective and antiinflammatory activities of the methanolic extract of *A. scrobicularis* herbs were reported; *A. scrobicularis* was toxicologically safe when orally taken and possessed very important hepatoprotective and antiinflammatory activities and it had the potential to be used in inflammatory and hepatic diseases [86]. As for the antimicrobial and antioxidant activities of methanol extracts of *A. cretica* subsp. *argaea* and *A*. fumariifolia, they showed 59.10% and 55.41% inhibition against linoleic acid oxidation, respectively. The study demonstrated that the methanol extracts of *A. fumariifolia* and *cretica A*. subsp. *argaea* had strong antibacterial activity against many tested bacteria (14 mm and 15 mm inhibition zone against *B. cereus*) [87].

The literature comparison of this study showed that similar compounds were found at different rates. However, more terpene components (74.70%) were characterized in this work. In addition, borneol was detected as major compound in the EO of the *C. tinctoria* var. *tinctoria* that may be used as taxonomical marker for the future classification of the *C. tinctoria* var. *tinctoria*. The variations in the volatile organic compounds on aerial parts of *C. tinctoria* var. *tinctoria* with other species may be due to environmental and analysis conditions. Thus, it could be pointed out that qualitative and quantitative results of this study were quite different from the previous reports.

### 3.1. Antimicrobial activity of EO and solvent extracts

The antimicrobial properties of the EO, *n-*hexane, acetonitrile, methanol, and aqueous extracts of *C. tinctoria* var. *tinctoria* were tested by an in vitro agar-well diffusion method using *P. aeruginosa*, *Y. pseudotuberculosis*, *E. coli*, *E. faecalis*, *B. cereus*, *S. aureus*, *M. smegmatis*, *C. tropicalis*, *S. cerevisiae*, and *C. albicans* (Table 2). After the inhibition diameters were observed in mm, the MIC values (μg/mL) were calculated [76–77] (Table 2). EO of *C. tinctoria* var. *tinctoria* was the most active against *M. smegmatis* with 15 mm inhibition (MIC 372.5 μg/mL). The acetonitrile extract of *C. tinctoria* var. *tinctoria* showed the best zone diameters as 14 mm, 15 mm, and 12 mm against *S. aureus*, *B. cereus*, and *M. smegmatis* with MIC values of 274 μg/mL, 274 μg/mL, and 548 μg/mL, respectively. EO and solvent extracts were more active for the gram-positive bacteria, while EO extract was only active for the gram-negative *Y. pseudotuberculosis*. These antimicrobial activities indicate the presence of active components in these extracts. None of the extracts were active against *E. coli*, *P. aeruginosa*, and *E. faecalis*. *n-*Hexane, methanol, and water extracts of *C. tinctoria* var. *tinctoria* were not active against tested fungi. No correlation was observed between solvent polarities and antimicrobial activity.

In the previous antimicrobial evaluation of *Anthemis species*; essential oils of *A. pseudocotula A. pectinata* var. *pectinata* and *A. dipsacea* were reported against eight microorganisms but did not affect the growth of *E. faecalis*, *Enterobacter cloacae*, *Salmonella thyphimurium*, and *Staphylococcus epidermidis*. When compared with standard antibiotics such as ceftazidime, sulbactam, ampicillin, and nystatin; the essential oils of *A. pseudocotula*, *A. pectinata* var. *pectinata*, and *A. dipsacea* at a concentration of 20 μL/disc had inhibitory effect on *E. coli*, *P. aeruginosa*, and *S. aureus* [19]. EO of *A. cretica* subsp*. messanensis* showed quite a good antimicrobial activity towards *E. coli*, *Streptococcus faecalis*, and *S. epidermidis* with MIC values of 25 μg/mL, 25 μg/mL, and 12.5 μg/mL, respectively [34]. Antimicrobial activities of aerial parts methanolic extract and essential oil of *A. stiparum* subsp. *sabulicola* were reported, and methanol extract displayed better antimicrobial activity than EO of *A*. stiparum subsp. *sabulicola*, being active against *S. aureus* and *Bacillus subtilis*, with MIC of 1.56 mg/mL [82]. Methanol extract of *A. fumariifolia and A. cretica* subsp. *argaea* were reported against 13 bacteria and two yeasts. Test results showed that the methanol extract had great potential of antibacterial activity against many bacteria which were tested. The inhibition zones for bacterial strains were found to be in the range of 6–14 mm and 7–15 mm, respectively. Nevertheless, they had no inhibitory effect on *S. cerevisiae* and *C. albicans* [87].

### 3.2. Lipase enzyme activity of EO and solvent extracts

The literature has shown that some of the common natural sources with lipase inhibitors contain active ingredients including polyphenols, flavonoids, terpenoids, and other active ingredients [35]. Terpenoids were the major constituents of the EO; thus, we investigated the lipase activity of EO and solvent extracts of *C. tinctoria* var. *tinctoria*. Essential oil and solvent extracts of *C. tinctoria* var. *tinctoria* were evaluated for lipase enzyme inhibition activities compared with orlistat as positive control (IC_50_: 13.49 ± 1.2262 μg/mL). The highest activity was found in the methanol extract (IC_50_: 48.73 ± 2.8265 μg/mL). Afterwards, the best activities were determined with IC_50_ values of 52.60 ± 3.7526 μg/mL, 59.80 ± 4.3285 μg/mL, and 68.28 ± 3.1215 μg/mL in acetonitrile, essential oil, and *n-*hexane, respectively (Figure). The lowest activity was found in the water extract (IC_50_: 99.5 ± 5.5678). No correlation was observed between solvent polarities and lipase activity. In another study, enzyme inhibitions of ethyl acetate, methanol, and water extracts of *A. chia* L. flowers were reported. MeOH extract of it showed the highest activity in tyrosinase inhibitory and *α-*amylase activity with 290.22 mg kojic acid equivalents (KAEs)/g extract and 413.66 mg acarbose equivalents (ACEs)/g extract, respectively [88]. In a study, key enzyme inhibitory potentials for the ethyl acetate, methanol, and aqueous extracts obtained from aerial parts of *A. cretica* subsp. *tenuiloba* and *A*. tinctoria var. *pallida* were mentioned. Ethyl acetate and methanol extracts showed potent activity against AChE with the highest activity observed for methanol extract (3.28 ± 0.43 mg GALAE/g) of *A*. tinctoria var. *pallida* and ethyl acetate extract of *A. cretica* subsp. *tenuiloba* (4.68 ± 0.21 mg GALAE/g). In case of BChE inhibitions of extracts; ethyl acetate extract of *A*. tinctoria var. *pallida* (3.48 ± 0.21 mg GALAE/g) and ethyl acetate (2.51 ± 0.34 mg GALAE/g), and methanol (1.15 ± 0.05 mg GALAE/g) extract of *A. cretica* subsp. *tenuiloba* were found to be more promising. Furthermore, enzyme inhibitory effects against *α*-glucosidase and tyrosinase were given, as well [89].

## 4. Conclusion

The composition of the EO obtained from aerial part of *C. tinctoria* var. *tinctoria* characterized and lipase enzyme and antimicrobial activities for the EO and solvent extracts were investigated for the first time. Monoterpenes were the main chemical class in the EO. Borneol (18.1%), camphor (14.9%), and ***β****-*pinene (11.3%) were the major components in the EO of *C. tinctoria* var. *tinctoria*. The EO showed the best activity against *M. smegmatis* (372.5 μg/μL MIC value). The acetonitrile extract was the most active against the *S. aureus* and *B. cereus* (274 μg/mL MIC value). The best activity for the lipase enzyme inhibitory of EO and solvent extracts (*n-*hexane, acetonitrile, methanol, and water) was found to be methanol extract with 48.73 μg/mL IC_50_ value. Therefore, the overall results of observed lipase enzyme and antimicrobial activities suggest that EO and solvent extracts of *C. tinctoria* var. *tinctoria* could be promising for pharmaceutical and other industrial applications. In a further study, Bio-guided activity isolation and purification could be carried out on *C. tinctoria* var. *tinctoria* for the bioavailability.

## Figures and Tables

**Figure:**
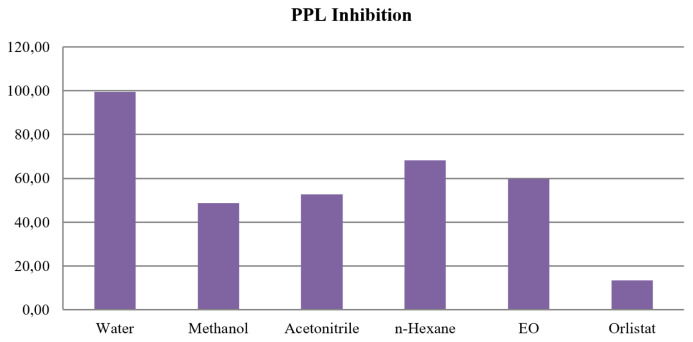
Porcine pancreatic lipase (PPL) inhibitory effect assay of essential oil and the solvent extracts (water, methanol, acetonitrile, and *n*-hexane) of *C. tinctoria* var. *tinctoria*. PPL inhibitory (IC50 = μg/mL ± SD) (Values are mean ± SD, n = 3), *p ≤ 0.05

**Table 1:** Essential oil components from aerial parts of *C. tinctoria* var. *tinctoria*.

Compounds	RI*	RI^a^	(%)^c^
Pentanal	732 [52]	733	0.05
2-Ethyl furan	728 [53]	735	0.08
1-Octene	794 [54]	801	0.76
Hexanal	801 [41]	807	0.97
2*E*-Hexenal	852 [55]	857	0.30
*n*-Hexanol	863 [41]	868	0.07
1-Nonene	893 [56]	896	0.10
Heptanal	901 [41]	906	0.07
Tricylene	929 [57]	930	0.27
*α*-Pinene	940 [58]	941	6.35
Camphene	953 [59]	956	10.69
Verbenene	961 [41]	960	0.12
Sabinene	978 [60]	978	0.72
*β*-Pinene	974 [41]	980	11.26
2-Pentyl furan	990 [61]	993	0.58
Octanal	1003 [56]	1003	0.18
*α*-Phellandrene	1006 [59]	1008	0.07
*α*-Terpinene	1018 [62]	1020	0.56
*o*-Cymene	1022 [41]	1026	0.15
Limonene	1031 [63]	1032	3.92
Eucalyptol	1034 [63]	1036	6.67
*Z*-*β*-Ocimene	1046 [64]	1047	0.76
*γ*-Terpinene	1060 [51]	1061	1.06
*α***-**Terpinolene	1086 [41]	1091	0.18
Linalool	1095 [41]	1097	0.59
Nonanal	1100 [41]	1103	0.84
1-Terpineol	1130 [41]	1133	0.08
Verbenol	1130 [41]	1134	0.14
Camphor	1146 [65]	1150	14.90
2*E*-Nonenal	1157 [66]	1159	0.18
Borneol	1169 [67]	1171	18.11
*α*-Terpineol	1191[68]	1194	0.83
*β*-Cyclocitral	1220 [31]	1224	0.03
Perillaldehyde	1263 [69]	1268	0.09
Bicycloelemene	1330 [70]	1336	0.06
Neryl acetate	1359 [41]	1361	0.12
*α*-Copaene	1382 [71]	1381	0.31
*β*-Caryophyllene	1417 [41]	1420	1.31
Neryl acetone	1434 [41]	1438	2.41
*α*-Humulene	1452 [41]	1458	0.43
Valencene	1489 [72]	1486	6.37
*β*-Bisabolene	1505 [41]	1510	2.24
*β*-Curcumene	1514 [41]	1519	0.04
Spathulenol	1577 [41]	1581	1.40
Guaiol	1601 [41]	1604	2.53
*γ*-Eudesmol	1635 [73]	1640	0.08
Cubenol	1645 [41]	1649	0.61
*α*-Bisabolol	1685 [41]	1689	0.01
Pentadecanal	1710 [74]	1710	0.04
Palmitic acid	1966 [75]	1962	0.00
Tricosane	2300 [41]	2296	0.15
Chemical classes	%^c^	NC^d^	
Monoterpenes	36.11	13	
Oxygenated Monoterpen	23.22	7	
Sesquiterpenes	10.75	7	
Oxygenated Sesquiterpen	4.62	5	
Terpene related	20.51	2	
Aldehyde	2.69	9	
Alcohol	0.07	1	
Aliphatic hydrocarbons	1.00	3	
Aromatic hydrocarbons	0.65	2	
Esters	0.12	1	
Acids	0.01	1	
Total	99.79	51	

* Literature RI values;

a Retention index calculated from retention times relative to that of *n*-alkane series (C_6_–C_30_);

b HD: Hydrodistillation;

c %: Percentages obtained by FID peak-area normalization;

d NC: Number of compounds.

**Table 2:** Antimicrobial activity of the EO and solvent extracts of *C. tinctoria* var. *tinctoria*.

Sample extracts	Const. (μg/mL)		Microorganisms, inhibition zone (mm), and minimal inhibition concentration (MIC, μg/mL)									
			Gram (−)			Gram (+)			No Gr.	Fungi		
			*Ec*.	*Yp*.	*Pa*.	*Ef*.	*Sa*.	*Bc*.	*Ms*.	*Ca*.	*Ct*.	*Sc*.
EO	59600	mm	-	8	-	-	6	10	15	8	8	10
		MIC	-	2980	-	-	2980	1490	372.5	2980	2980	1490
*n*-Hexane	72200	mm	-	-	-	-	6	-	-			
		MIC	-	-	-	-	3610	-	-	-	-	-
Acetonitrile	43900	mm	-	-	-	-	14	15	12	6	6	6
		MIC	-	-	-	-	274	274	548	2195	2195	2195
Methanol	52340	mm	-	-	-	-	14	12	12			
		MIC	-	-	-	-	3271	6542	6542	-	-	-
Water	43500	mm	-	-	-	-	-	-	10			
		MIC	-	-	-	-	-	-	1087	-	-	-
Amp.	10	mm	10	10	18	10	10	15				
		MIC	10	18	128	35	10	15				
Strep.	10	mm							35			
		MIC							4			
Flu	5	mm								25	25	25
		MIC								<8	<8	<8

Ec*: Escherichia coli*, Yp: *Yersinia pseudotuberculosis*, Pa: *Pseudomonas aeruginosa*, Sa: *Staphylococcus aureus*, Ef: *Enterococcus faecalis*, Bc: *Bacillus cereus* 702 Roma, Ms: *Mycobacterium smegmatis*, Ca: *Candida albicans*, Sc: *Saccharomyces cerevisiae*, Ct: *Candida tropicalis*, Amp.: Ampicillin, Strep.: Streptomycin, Flu.: Fluconazole, (−): no activity of test concentrations

## References

[1] Grierson AJC Yavin Z Flora of Turkey and the East Aegean Islands Davis P Edinburgh Edinburgh University Press 1975 174 221

[2] Greuter W Oberprieler C Vogt R The Euro+Med treatment of Anthemideae (Compositae)–generic concepts and required new names Willdenowia 2003 33 37 43 10.3372/wi.33.33102

[3] Lo Presti RM Oppolzer S Oberprieler CA Molecular phylogeny and a revised classification of the Mediterranean genus Anthemis s.l. (Compositae, Anthemideae) based on three molecular markers and micromorphological characters Taxon 2010 59 1441 1456 10.1002/tax.595010

[4] Özbek MU Vural M Synopsis of the Genus Cota (Anthemideae, Asteraceae) in Turkey Communications Faculty of Sciences University of Ankara Series C Biology 2020 29 2 275 299

[5] The Plant List 2021 A working list of all plants Flowering Plants [online] Website http://www.theplantlist.org/tpl1.1/record/gcc-33458 accessed 19 October 2021

[6] Oberprieler C Vogt R Watson LE Tribe Anthemideae Cass Kadereit JW Jeffrey C Kubitzki’s The families and genera of vascular plants Springer-Verlag Berlin: Heidenberg 2007 342 374

[7] Özbek MU Gay Cota J Güner A A Checklist of the Flora of Turkey (Vascular Plants), Nezahat Gökyiğit Botanic Garden and Floristics Research İstanbul, Turkey Society Publication 2012 146 148

[8] Özbek MU Vural M Daşkın R A new species of the genus Cota (Asteraceae) from Uludağ, Turkey Turkish Journal of Botany 2011 35 331 336 10.3906/bot-1002-27

[9] Shawahna R Jaradat NA Ethnopharmacological survey of medicinal plants used by patients with psoriasis in The West Bank of Palestine BMC Complementary and Alternative Medicine 2017 17 1 4 28049474 10.1186/s12906-016-1503-4 PMC5209870

[10] Al-Snafi AE Medical importance of Anthemis nobilis (Chamaemelum nobile)-A review Asian Journal of Pharmaceutical Science and Technology 2016 6 2 89 95

[11] Ugurlu E Secmen O Medicinal Plants popularly used in the villages of yunt mountain (Manisa-Turkey) Fitoterapia 2008 79 2 126 131 10.1016/j.fitote.2007.07.016 17878061

[12] Honda G Yesilada E Tabata M Sezik E Fujita T Traditional medicine in Turkey. VI. Folk Medicine in west Anatolia: Afyon, Kütahya, Denizli, Muğla, Aydin provinces Journal of Ethnopharmacology 1996 53 2 75 87 10.1016/S0378-8741(96)01426-2 8844462

[13] Cakilcioglu U Khatun S Turkoglu I Hayta S Ethnopharmacological survey of medicinal plants in Maden (Elazig-Turkey) Journal of Ethnopharmacology 2011 137 469 486 10.1016/j.jep.2011.05.046 21704144

[14] Papaioannoua P Lazaria D Kariotib A Soulelesa C Heilmannc J Phenolic compounds with antioxidant activity from Anthemis tinctoria L (Asteraceae) Zeitschrift für Naturforschung C 2007 62c 326 330 10.1515/znc-2007-5-603 17708435

[15] Conforti F Menichini F Formisano C Rigano D Senatore F Anthemis wiedemanniana essential oil prevents lps-ınduced production of NO in RAW 264.7 macrophages and exerts antiproliferative and antibacterial activities in vitro Natural Product Research 2012 26 17 1594 1601 10.1080/14786419.2011.585988 22124231

[16] Afifi FU Kasabri V Pharmacological and phytochemical appraisal of selected medicinal plants from Jordan with claimed antidiabetic activities Scientia Pharmaceutica 2013 81 4 889 932 10.3797/scipharm.1212-20 24482764 PMC3867248

[17] De Mieri M Monteleone G Ismajili I Kaiser M Hamburger M Antiprotozoal activity-based profiling of a dichloromethane extract from Anthemis nobilis flowers Journal of Natural Products 2017 80 2 459 470 10.1021/acs.jnatprod.6b00980 28116906

[18] Karim A Berrabah M Mekhfi H Ziyyat A Legssyer A Effect of essential oil of Anthemis mauritiana Maire & Sennen Flowers on ıntestinal smooth muscle contractility Journal of Smooth Muscle Research 2010 46 1 65 75 10.1540/jsmr.46.65 20383035

[19] Kurtulmuş A Fafal T Mert T Saglam H Kivcak B Chemical composition and antimicrobial activity of the essential oils of three Anthemis species from Turkey Chemistry of Natural Compounds 2009 45 6 900 904

[20] Baser KHC Demirci B Iscan G Hashimoto T Demirci F The essential oil constituents and antimicrobial activity of Anthemis aciphylla Boiss. var. discoidea Boiss Chemical and Pharmaceutical Bulletin 2006 54 2 222 10.1248/cpb.54.222 16462068

[21] Saroglou V Dorizas N Kypriotakis Z Skaltsa HD Analysis of the essential oil composition of eight Anthemis species from Greece Journal of Chromatography A 2006 1104 1–2 313 322 10.1016/j.chroma.2005.11.087 16359681

[22] Sajjadi SE Mehregan I Volatile constituents of flowers and leaves of Anthemis hyalina Chemistry of Natural Compounds 2006 42 531 533

[23] Holla M Svajdlenka E Vaverska S Zibrunova B Tekel J Composition of the oil from the flowerheads of Anthemis tinctoria L. cultivated in Slovak Republic Journal of Essential Oil Research 2000 12 6 714 716 10.1080/10412905.2000.9712198

[24] Baser KHC Ozek T Demirci F Boydag I The essential oil of Anthemis cretica L. subsp. leucanthemoides (Boiss.) Grierson Acta Pharmaceutica Turcica 2002 44 3 189 194

[25] Vujisic LJ Vuckovic I Tesevic V Dokovic D Ristic MS Comparative examination of the essential oils of Anthemis ruthenica and A. arvensis wild-growing in Serbia Flavour and Fragrance 2006 21 3 458 461 10.1002/ffj.1681

[26] Kivcak B Mert T Saglam H Ozturk T Kurkcuoglu M Chemical composition and antimicrobial activity of the essential oil of Anthemis wiedemanniana from Turkey Chemistry of Natural Compounds 2007 43 47 51

[27] Grace MH Screening of selected indigenous plants of Lebanon for antimicrobial activity Phytotherapy Research 2002 16 183 185 11933125

[28] Bulatovic VM Vajs VE Macura N Juranic N Milosavljevic SM Highly oxygenated guaianolides from Anthemis carpatica Journal of Natural Products 1997 60 12 1222 1228 10.1021/np970185w

[29] Uzel A Guvensen A Cetin E Chemical composition and antimicrobial activity of the essential oils of Anthemis xylopoda O. Schwarz from Turkey Journal of Ethnopharmacology 2004 95 2–3 151 154 10.1016/j.jep.2004.06.034 15507328

[30] Bulatovic VM Menkovic NR Nebojsa R Vajs VE Milosavljevic SM Essential oil of Anthemis montana Journal of Essential Oil Research 1998 10 2 223 226 10.1080/10412905.1998.9700887

[31] Javidnia K Miri R Kamalinejad M Sarkarzadeh H Jamalian A Chemical composition of the essential oils of Anthemis altissima L. grown in Iran Flavour and Fragrance 2004 19 3 213 216 10.1002/ffj.1277

[32] Pavlovic M Kovacevic N Tzakou O Couladis M Essential oil composition of Anthemis triumfetti (L.) DC Flavour and Fragrance 2006 21 2 297 299 10.1002/ffj.1592

[33] Rezaee MB Jaimand K Assareh MH Chemical constituents of the leaf and flower oils from Anthemis altissima L. var. altissima from Iran Journal of Essential Oil Research 2006 18 2 152 153 10.1080/10412905.2006.9699049

[34] Riccobono L Maggio A Bruno M Spadaro V Raimondo F Chemical composition and antimicrobial activity of the essential oils of some species of Anthemis sect. Anthemis (Asteraceae) from Sicily Natural Product Research 2017 31 23 2759 2767 10.1080/14786419.2017.1297444 28278620

[35] Liu TT Liu XT Chen QX Shi Y Lipase ınhibitors for obesity: A Review Biomedicine & Pharmacotherapy 2020 128 110314 10.1016/j.biopha.2020.110314 32485574

[36] Emir A Emir C Quantification of phenolic compounds of Anthemis tinctoria L. var tinctoria L by LC-ESI-MS/MS and determination of biological activities of the plant Gumushane University Journal of Science Institute 2020 10 4 996 1006

[37] Shamloo S Jafari Marandi S Tajadod G Majd A Rahimi R Cytotoxic effect of hydroalcoholic extract of Cota tinctoria (L.) J. Gay on AGS and Hep-G2 cancer cell lines Boletín Latinoamericano y del Caribe de Plantas Medicinales y Aromáticas 2022 21 1 108 122

[38] Eser F Sahin Yaglioglu A Dolarslan M Aktas E Onal A Dyeing, fastness, and cytotoxic properties, and phenolic constituents of Anthemis tinctoria var. tinctoria (Asteraceae) The Journal of The Textile Institute 2017 108 9 1489 1495

[39] Erik İ Kılıç G Öztürk E Karaoğlu ŞA Yaylı N Chemical composition, antimicrobial, and lipase enzyme activity of essential oil and solvent extracts from Serapias orientalis subsp. orientalis Turkish Journal of Chemistry 2020 44 6 1655 1662 https://doi:10.3906/kim-2005-51 33488260 10.3906/kim-2005-51 PMC7763115

[40] Yayli N Fandakli S Korkmaz B Barut B Renda G Biological evaluation (antimicrobial, antioxidant, and enzyme inhibitions), total phenolic content and volatile chemical compositions of Caucasalia macrophylla (M. Bieb.) B. Nord. (Asteraceae) Journal Essential Oil-Bearing Plants 2018 21 5 1359 1373

[41] Adams RP Identification of Essential Oil Components by Gas Chromatography/mass Spectrometry Allured Publishing Corporation Carol Stream, IL 2007

[42] Ucuncu O Kahriman N Terzioglu S Karaoglu SA Yayli N Composition and antimicrobial activity of the essential oils from flowers of Senecio othonnae, S. racemosus, and S. nemorensis Natural Product Communications 2010 5 5 831 834 10.1177/1934578X1000500531 20521557

[43] Yayli N Yasar A Yilmaz Iskender N Yayli N Cansu TB Chemical constituents and antimicrobial activities of the essential oils from Sedum pallidum var. bithynicum and S. spurium grown in Turkey Pharmaceutical Biology 2010 48 2 191 194 10.3109/13880200903074627 20645839

[44] Ucuncu O Yayli N Volga C Yayli N Terzioglu S Chemical composition of essential oils from flower, leaf and stem of Aquilegia olympica grown in Turkey Asian Journal of Chemistry 2009 21 8 6569 6574

[45] Erik I Kılıc G Korkmaz B Fandaklı S Alpay Karaoğlu S Volatile constituents and antimicrobial activities of Vinca major L. subsp. hirsuta (Boiss) stearn grown in Turkey Journal of Research in Pharmacy 2021 25 5 581 588 10.29228/jrp.49

[46] Sparkman OD Identification of essential oil components by gas chromatography/quadrupole mass spectroscopy Journal of the American Society for Mass Spectrometry 2005 16 11 1902

[47] Andriamaharavo N Retention Data NIST Mass Spectrometry Data Center NIST Mass Spectrometry Data Center 2014

[48] Yayli N Gülec C Üçüncü O Yaşar A Ülker S Composition and antimicrobial activities of volatile components of Minuartia meyeri Turkish Journal of Chemistry 2006 30 1 71 76

[49] Cansu TB Yaylı B Özdemir T Batan N Karaoğlu ŞA Antimicrobial activity and chemical composition of the essential oils of mosses (Hylocomium splendens (Hedw.) Schimp. and Leucodon sciuroides (Hedw.) Schwägr.) growing in Turkey Turkish Journal of Chemistry 2013 37 2 213 219

[50] Kan Y Uçan US Kartal M Altun ML Aslan S GC-MS analysis and antibacterial activity of cultivated Satureja cuneifolia Ten. essential oil Turkish Journal of Chemistry 2006 30 2 253 259

[51] Adams RP Morris JA Pandey RN Schwarzbach AE Cryptic speciation between Juniperus deltoides and Juniperus oxycedrus (Cupressaceae) in the Mediterranean Biochemical Systematics and Ecology 2005 33 8 771 787 10.1016/j.bse.2005.01.001

[52] Ozel MZ Gogus F Lewis AC Comparison of direct thermal desorption with water distillation and superheated water extraction for the analysis of volatile components of Rosa damascena Mill. using GCxGC-TOF/MS Analytica Chimica Acta 2006 566 2 172 177 10.1016/j.aca.2006.03.014

[53] Jordán MJ Margaría CA Shaw PE Goodner KL Aroma active components in aqueous kiwi fruit essence and kiwi fruit puree by GC-MS and multidimensional GC/GC-O Journal of Agricultural and Food Chemistry 2002 50 19 5386 5390 10.1021/jf020297f 12207479

[54] Insausti K Goñi V Petri E Gorraiz C Beriain MJ Effect of weight at slaughter on the volatile compounds of cooked beef from Spanish cattle breeds Meat Science 2005 70 1 83 90 10.1016/j.meatsci.2004.12.003 22063283

[55] Siegmund B Murkovic M Changes in chemical composition of pumpkin seeds during the roasting process for production of pumpkin seed oil Food Chemistry 2004 84 3 367 374 10.1016/S0308-8146(03)00241-3

[56] Flamini G Tebano M Cioni PL Bagci Y Dural H A multivariate statistical approach to Centaurea classification using essential oil composition data of some species from Turkey Plant Systematics and Evolution 2006 261 1–4 217 228 10.1007/s00606-006-0448-3

[57] Jordan MJ Martinez RM Goodner KL Baldwin EA Sotomayor JA Seasonal variation of Thymus hyemalis Lange and Spanish Thymus vulgaris L. essential oils composition Industrial Crops and Products 2006 24 3 253 263 10.1016/j.indcrop.2006.06.011

[58] Bouzouita N Kachouri F Hamdi M Chaabouni MM Antimicrobial activity of essential oils from Tunisian aromatic plants Flavour and Fragrance 2003 18 5 380 383 10.1002/ffj.1200

[59] Lopes D Strobl H Kolodziejczyk P 14-Methylpentadecano-15-lactone (Muscolide): a new macrocyclic lactone from the oil of Angelica archangelica L Chemistry and Biodiversity 2004 1 12 1880 1887 10.1002/cbdv.200490144 17191826

[60] Flamini G Tebano M Cioni PL Volatiles emission patterns of different plant organs and pollen of Citrus limon Analytica Chimica Acta 2007 589 1 120 124 10.1016/j.aca.2007.02.053 17397661

[61] Javidnia K Miri R Javidnia A Constituents of the essential oil of Scabiosa flavida from Iran Chemistry of Natural Compounds 2006 42 5 529 530 10.1007/s10600-006-0206-3

[62] Kitic D Palic R Ristic M Sojanovic G Jovanovic T The volatile constituents of Calamintha sylvatica Bromf. subsp. sylvatica Flavour and Fragrance 2001 16 4 257 258 10.1002/ffj.995

[63] Yáñez X Pinzón ML Solano F Sánchez LR Chemical composition of the essential oil of Psidium caudatum McVaugh Molecules 2002 7 9 712 716 10.3390/70900712

[64] Lopez Arze JB Collin G Garneau FX Jean FI Gagnon H Essential oils from Bolivia. II. Asteraceae: Ophryosporus heptanthus (Wedd.) H. Rob. et King Journal of Essential Oil Research 2004 16 4 374 376 10.1080/10412905.2004.9698747

[65] García D Alvarez A Tornos P Fernandez A Sáenz T Gas chromatographic-mass spectrometry study of the essential oils of Pimenta racemosa var. terebinthina and P. racemosa var. grisea Zeitschrift für Naturforschung C 2002 57c 449 451 10.1515/znc-2002-5-608 12132683

[66] Utsunomia H Kawata J Chanoki W Shirakawa N Miyazawa M Components of Essential Oil from Woods of Prunus mume Sieb. at Zucc Journal of Oleo Science 2005 54 11 609 612 10.5650/jos.54.609

[67] Daferera DJ Ziogas BN Polissiou MG The effectiveness of plant essential oils on the growth of Botrytis cinerea, Fusarium sp. and Clavibacter michiganensis subsp. michiganensis Crop Protection 2003 22 1 39 44 10.1016/S0261-2194(02)00095-9

[68] Sibanda S Chigwada G Poole M Gwebu ET Noletto JA Composition and bioactivity of the leaf essential oil of Heteropyxis dehniae from Zimbabwe Journal of Ethnopharmacolgy 2004 92 1 107 111 10.1016/j.jep.2004.02.010 15099856

[69] Liu JM Nan P Tsering Q Tsering T Bai ZK Volatile constituents of the leaves and flowers of Salvia przewalskii Maxim. from Tibet Flavour and Fragrance 2006 21 3 435 438 10.1002/ffj.1607

[70] Silva DB Pott A Oliveira DCR Analyses of the headspace volatile constituents of aerial parts (leaves and stems), flowers and fruits of Bidens gardneri Bak. and Bidens sulphurea (Cav.) Sch. Bip. using solid-phase microextraction Journal of Essential Oil Research 2010 22 6 560 563 10.1080/10412905.2010.9700400

[71] Dehghan G Solaimanian R Shahverdi AR Amin G Abdollahi M Chemical composition and antimicrobial activity of essential oil of Ferula szovitsiana D.C Flavour and Fragrance 2007 22 3 224 227 10.1002/ffj.1789

[72] Tzakou O Couladis M The essential oil of Micromeria graeca (L.) Bentham et Reichenb. growing in Greece Flavour and Fragrance 2001 16 2 107 109 10.1002/ffj.955

[73] Farsam H Amanlou M Taghi-Cheetsaz N Amin GR Saledi-Sormaghi MH Essential oil of Chimonanthus fragrans flowers Population of Tehran DARU Journal of Pharmaceutical Sciences 2007 15 3 129 131

[74] Javidnia K Miri R Banani A Volatile oil constituents of Haplophyllum tuberculatum (Forssk.) A. Juss. (Rutaceae) from Iran Journal of Essential Oil Research 2006 18 4 355 356 10.1080/10412905.2006.9699111

[75] Fokialakis N Melliou E Magiatis P Harvala C Mitaku S Composition of the steam volatiles of six Euphorbia spp. from Greece Flavour and Fragrance 2003 18 1 39 42 10.1002/ffj.1148

[76] Barry AL Standards NCfCL Methods for determining bactericidal activity of antimicrobial agents: approved guideline National Committee for Clinical Laboratory Standards Wayne, PA 1999

[77] Woods GL Susceptibility testing of mycobacteria, nocardiae, and other aerobic actinomycetes Approved Standard M24 A2 2011 31 5 31339680

[78] Sener SO Ozgen U Kanbolat S Korkmaz N Badem M Investigation of therapeutic potential of three endemic Cirsium species for global health problem obesity South African Journal of Botany 2021 141 243 254 10.1016/j.sajb.2021.05.004

[79] Pavlovic M Lakusic D Kovacevic N Tzakou O Couladis M Comparative analysis of essential oils of six Anthemis taxa from Serbia and Montenegro Chemistry & Biodiversity 2010 7 5 1231 1244 10.1002/cbdv.200900156 20491079

[80] Veverkova S Holla M Haban M Otepka P Mikulasova M Qualitative properties and content of essential oil in the flowerheads of Anthemis tinctoria L Acta Horticulturae 2007 749 283 287 10.17660/ActaHortic.2007.749.36

[81] Raal A Kaur H Orav A Arak E Kailas T Content and composition of essential oils in some Asteraceae species Proceedings of the Estonian Academy of Sciences 2011 60 1 5563 10.3176/proc.2011.1.06

[82] Chemsa AE Zellagui A Ozturk M Erol E Ceylan O Chemical composition, antioxidant, anticholinesterase, antimicrobial and antibiofilm activities of essential oil and methanolic extract of Anthemis stiparum subsp sabulicola (Pomel) Oberpr Microbial Pathogenesis 2018 119 233 240 10.1016/j.micpath.2018.04.033 29684540

[83] Orhan I Deliorman-Orhan D Ozcelik B Antiviral activity and cytotoxicity of the lipophilic extracts of various edible plants and their fatty acids Food Chemistry 2009 115 2 701 705 10.1016/j.foodchem.2009.01.024

[84] Williams CA Greenham J Harborne JB The role of lipophilic and polar flavonoids in the classification of temperate members of the Anthemideae Biochemical Systematics and Ecology 2001 29 9 929 945 10.1016/S0305-1978(01)00039-4 11445294

[85] Zebarjad Z Farjam MH Phytochemical Composition and antimicrobial and antioxidant activity of essential oil of Anthemis fungosa Chemistry of Natural Compounds 2017 53 1 156 158 10.1007/s10600-017-1935-1

[86] Yusufoglu HS Alam A Salkini MAA Zaghloul AM Anti-inflammatory and hepatoprotective activities of methanolic extract of Anthemis scrobicularis herbs Pharmacognosy Journal 2014 6 3 55 61 10.5530/pj.2014.3.9

[87] Albayrak S Aksoy A Evaluation of antioxidant and antimicrobial activities of two endemic Anthemis species in Turkey Journal of Food Biochemistry 2013 37 6 639 645 10.1111/j.1745-4514.2012.00654.x

[88] Sarikurkcu C Anthemis chia: Biological capacity and phytochemistry Industrial Crops and Products 2020 153 112578 10.1016/j.indcrop.2020.112578

[89] Orlando G Zengin G Ferrante C Ronci M Recinella L Comprehensive chemical profiling and multidirectional biological investigation of two wild Anthemis species (Anthemis tinctoria var. pallida and A. cretica subsp. tenuiloba): focus on neuroprotective effects Molecules 2019 24 14 2582 2606 10.3390/molecules24142582 31315236 PMC6680454

